# Supplementary figures and images for: Molecular and phenotypic distinctions of macrophages in tolerant and susceptible to hypoxia rats
Source: PeerJ. 2023 Oct 10;11:e16052. doi: 10.7717/peerj.16052 (PMC10573310; doi:10.7717/peerj.16052)

## Slide 1
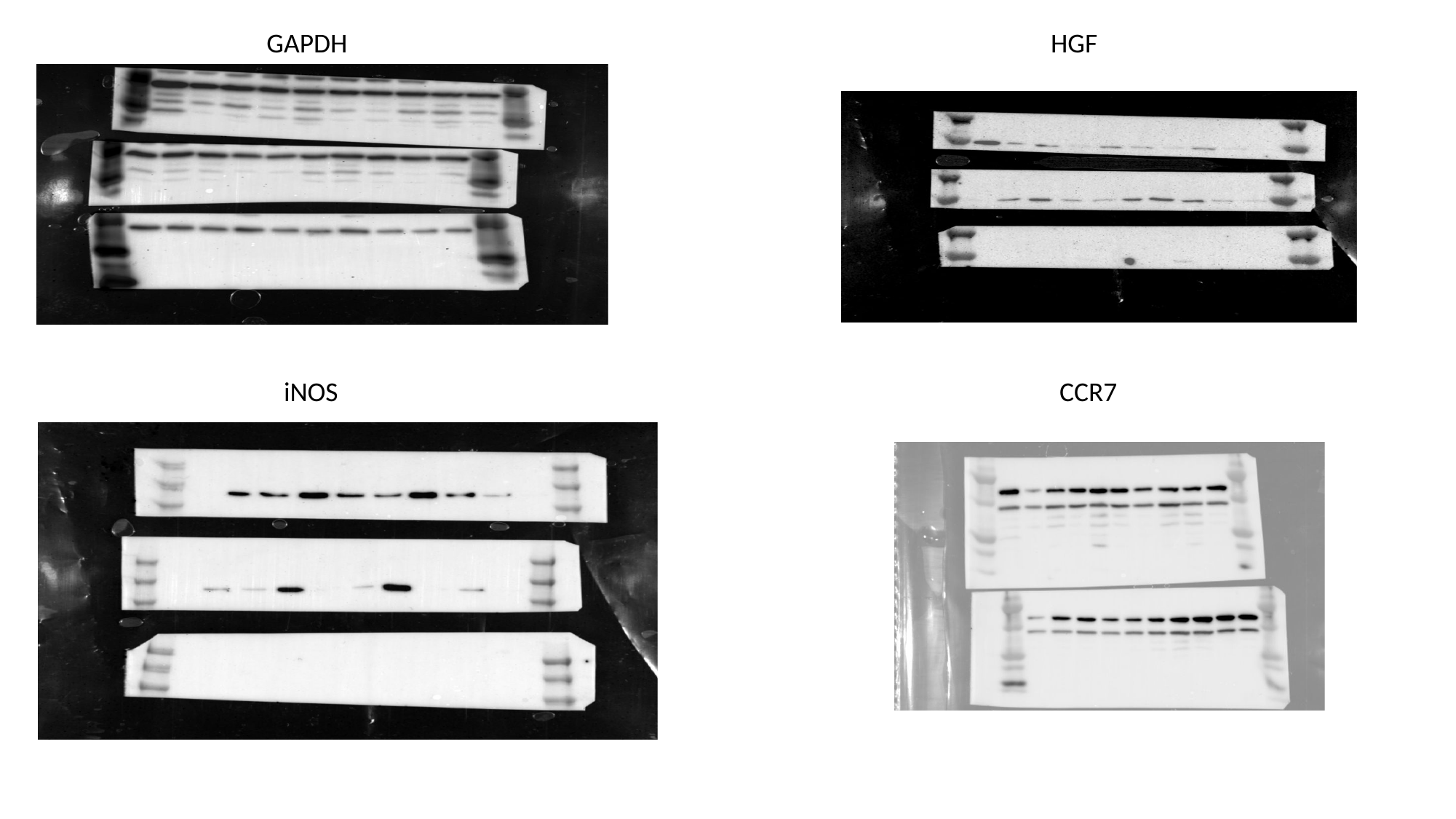

GAPDH
HGF
iNOS
CCR7

## Slide 2
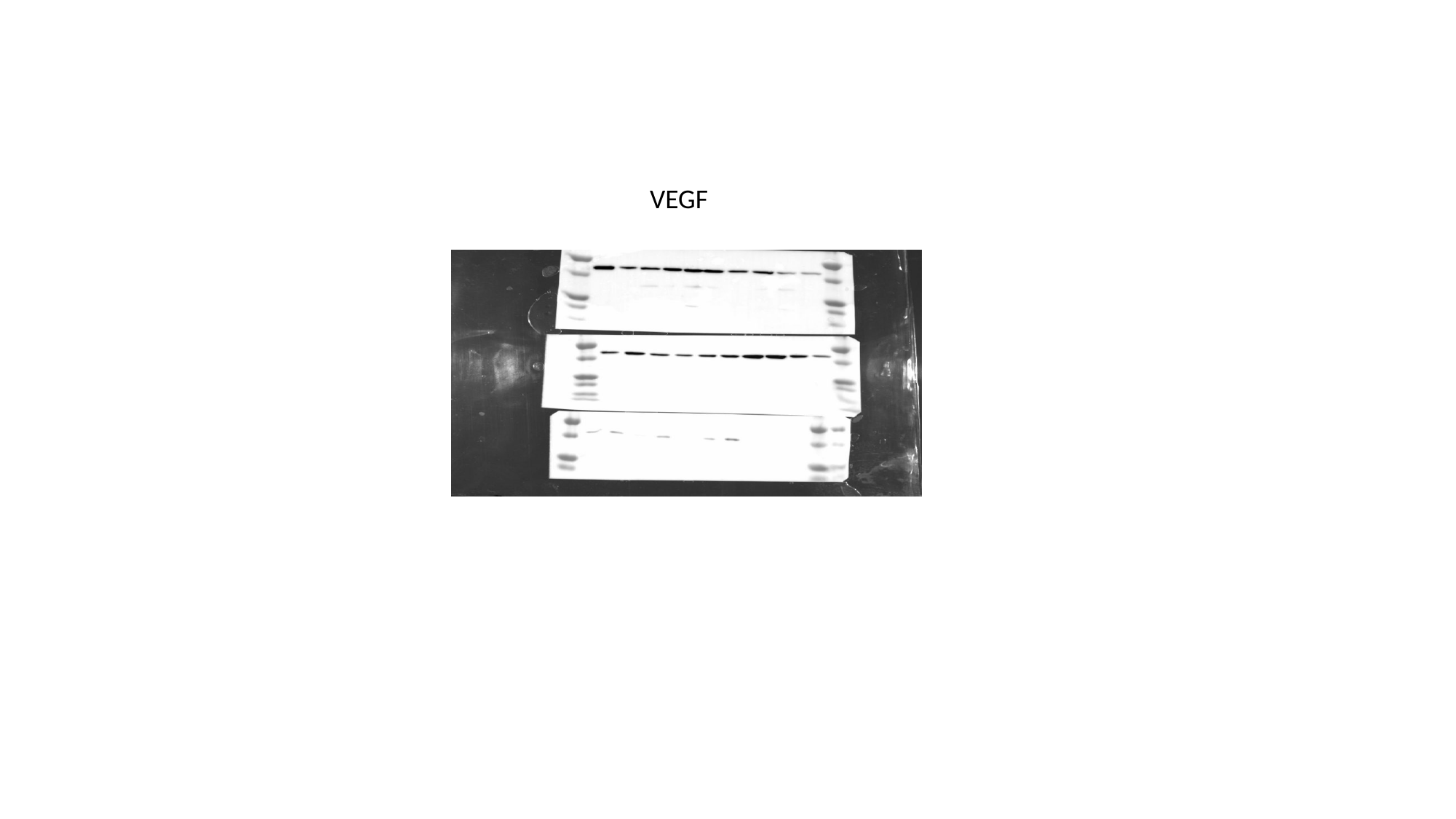

VEGF

Supplement: Supplemental Information 2 [file peerj-11-16052-s002.zip › WB membranes_Supplemental.pptx]
